# Clade 2.3.4.4b Highly Pathogenic Avian Influenza H5N1 Pathology in a Common Shorebird Species (Sanderling; *Calidris alba*) in Virginia, USA

**DOI:** 10.3390/ani15142057

**Published:** 2025-07-12

**Authors:** Victoria A. Andreasen, Emily G. Phillips, Aidan M. O’Reilly, C. Robert Stilz, Rebecca L. Poulson, Ruth Boettcher, John K. Tracey, Nicole M. Nemeth

**Affiliations:** 1 Southeastern Cooperative Wildlife Disease Study, University of Georgia, Athens, GA 30602, USA; tandreasen@uga.edu (V.A.A.); emilyphillips@uga.edu (E.G.P.); aidan.oreilly25@uga.edu (A.M.O.); robert.stilz@uga.edu (C.R.S.); rpoulson@uga.edu (R.L.P.); 2 Center for the Ecology of Infectious Diseases, University of Georgia, Athens, GA 30602, USA; 3 Warnell School of Forestry and Natural Resources, University of Georgia, Athens, GA 30602, USA; 4 Department of Pathology, University of Georgia, Athens, GA 30602, USA; 5 Virginia Department of Wildlife Resources, Machipongo, VA 23405, USA; ruth.boettcher@dwr.virginia.gov; 6 Virginia Department of Wildlife Resources, Verona, VA 24482, USA; john.tracey@dwr.virginia.gov

**Keywords:** shorebird, pathology, viral disease, morbidity, mortality, outbreak, tropism, North America, Atlantic coast

## Abstract

Anseriformes (waterfowl) and Charadriiformes (shorebirds) are well-recognized natural reservoirs of low pathogenic (LP) influenza A viruses (IAVs). Historically, LP IAVs circulate among healthy individuals during seasonal, and often transcontinental, migrations. However, following the introduction of clade 2.3.4.4b highly pathogenic (HP) A/Goose/Guangdong/1/1996 lineage H5 IAV to North America in 2021, countless wild birds succumbed to fatal infections across the Western Hemisphere. Due to their small size and cryptic plumage patterns, opportunities for carcass recovery and postmortem evaluation in sanderlings (*Calidris alba*) and other shorebirds are rare. A multispecies mortality event in coastal Virginia, USA, in March–April 2024 included sanderlings among other wild bird species. Nine sanderlings underwent postmortem evaluation and clade 2.3.4.4b H5 IAV RNA was detected in pooled oropharyngeal-cloacal swabs from 11/11 individuals by real-time reverse transcription polymerase chain reaction. Histopathology was similar to that in waterfowl and included necrosis in the pancreas and brain and less commonly in the gonad, adrenal gland, spleen, liver, and intestine. Immunohistochemistry revealed IAV antigen labeling in necrotic neurons of the brain (neurotropism) and epithelial cells of the pancreas, gonad, and adrenal gland (epitheliotropism). Describing HP IAV-attributed pathology in shorebirds is key to understanding ecoepidemiology and population health threats in order to further document and compare pathogenesis among avian species.

## Simple Summary

Waterfowl and shorebirds are established reservoirs of low pathogenic avian influenza viruses in nature. These viruses circulate in healthy birds during seasonal migration. However, in late 2021, a deadly Eurasian influenza virus strain, known as highly pathogenic H5 influenza virus, was introduced to Canada; it spread rapidly across North and South America and caused countless wild bird fatalities. The sanderling (*Calidris alba*) is a globally distributed, long-distance migratory shorebird that intermixes with other wild birds during migration when infection risk is high. We sought to address its susceptibility to highly pathogenic H5 influenza virus infection and disease during a mortality event in March–April 2024 in coastal Virginia, USA. We assessed pathology in sanderlings that died of influenza during this outbreak. Most had severe pancreatic and brain damage, which was less common in the digestive tract and other internal organs. The virus had an affinity for specific but widespread cells in the body (e.g., neurons in the brain; epithelial cells in numerous other organs). Thus, sanderlings are susceptible to rapidly fatal highly pathogenic H5 influenza virus infection. These results help gauge the population threat that these viruses pose to shorebirds, while recognizing potential environmental and human impacts of infected wild birds across the landscape.

## 1. Introduction

Numerous avian species in the orders Anseriformes (i.e., waterfowl) and Charadriiformes (i.e., shorebirds and gulls) are recognized as natural reservoirs of low pathogenic (LP) influenza A viruses (IAVs). The circulation and spread of LP IAVs among apparently healthy individuals during seasonal, and often transcontinental, migrations is a well-documented phenomenon of host–virus maintenance [1]. However, IAV infections in some shorebirds appear to be more localized and species-limited [2]. Furthermore, some species, such as the sanderling (*Calidris alba*), generally have low LP IAV detection prevalence (via cloacal swabs) and seroprevalence, and thus are considered relatively resistant to or less likely to be exposed to IAV infection [3]. For example, more commonly infected species, such as the ruddy turnstone (*Arenaria interpres*), could have increased exposure risk due to their nighttime preference for salt marsh habitats shared by waterfowl and gull breeding colonies, while sanderlings utilize sandy points and islands during this time [2]. The sanderling is a cosmopolitan and common shorebird species that utilizes every flyway in the Western Hemisphere and migrates thousands of kilometers (>10,000 km), from high Arctic breeding grounds to the southern tip of South America [4,5]. Both stopover and wintering sites are shared across seabirds and other coastal bird species, providing opportunities for pathogen transmission [3].

Following the introduction of clade 2.3.4.4b of highly pathogenic (HP) H5N1 IAV (hereafter, HP H5 IAV) to Northeastern Canada from Eurasia in December of 2021 [6], unprecedented numbers of wild birds of numerous species succumbed to fatal infection across North and South America [7,8], as well as more sensitive habitats such as Antarctica [9]. The most affected species groups recognized in the Western Hemisphere thus far are waterfowl, raptors, and seabirds, with few reports of fatal infections in shorebirds to date [3,7,8,10,11,12]. In the USA, among 13,225 wild and zoo bird detections confirmed as HP H5 IAV by the United States Department of Agriculture (USDA) National Veterinary Services Laboratory (NVSL), thus far, 3.25% are shorebirds (i.e., Charadriiformes) [8]. The diversity and magnitude of these HP H5 IAV-attributed wild bird mortalities, many undocumented or poorly understood, warrant continued assessment of mortality events to better understand potential impacts to population health. While shorebirds are recognized as subclinical reservoir hosts for LP IAVs [3,13], little is known about the health implications of HP H5 IAV in this group.

Our goal was to document HP H5 IAV-attributed pathology in sanderlings, including description of lesion patterns and viral antigen distribution in tissues. We performed postmortem evaluations on sanderling carcasses recovered during a multi-species outbreak in the mid-Atlantic coast of the USA during the spring migration and detected H5 IAV infection through molecular and immunohistochemical (IHC) assays. We also compared lesions caused by highly pathogenic avian influenza (HPAI)—that is, disease resulting from HP IAV infection—with those observed in other shorebird and wild bird species to better understand their varying susceptibility and potential roles in HP H5 IAV transmission and maintenance.

## 2. Materials and Methods

### 2.1. Mortality Event and Carcass Collection

**Figure 1:**
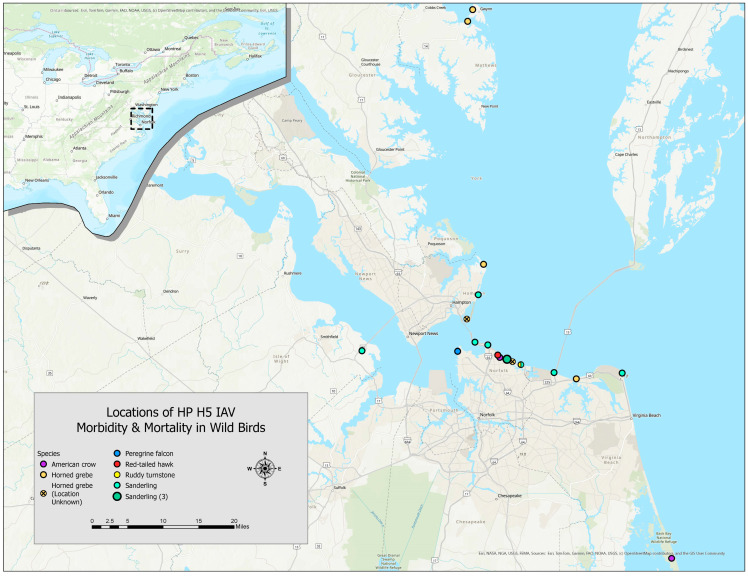
Locations in coastal Virginia, USA, where the morbidity and mortality of sanderlings and other wild birds in March–April 2024 were attributed to highly pathogenic H5N1 influenza A virus. The location of one sanderling was unspecified (not included on map), while three sanderlings were found in the same location (large green dot). Locations for two horned grebes (yellow dots with internal “X”) were estimated based on provided information (coordinates not available).

A wild bird mortality event from 7 to 29 March 2024, which lasted until mid-April 2024, spanned an area of ≥5652 km^2^, including multiple sites in four counties (Accomack, Mathews, Northumberland, and Middlesex) and three independent cities (Virginia Beach, Hampton, and Norfolk), primarily along coastal Virginia (Figure 1). Eleven sanderlings were among 34 wild birds collected after either being found dead or severely morbid and later died or were euthanized, with field estimates of hundreds of affected birds. Seven sanderlings were collected from Norfolk, two from Virginia Beach, and one from Hampton; the collection location of one sanderling was unspecified. Among the seven sanderlings observed while alive, some had neurologic signs (e.g., seizures). Carcasses or sick birds (later euthanized at rehabilitation clinics) were collected by members of the public, wildlife rehabilitator personnel/volunteers, or Virginia Department of Wildlife Resources personnel. Carcasses were stored at −20 °C for up to three weeks and submitted to the Southeastern Cooperative Wildlife Disease Study (SCWDS) at the University of Georgia (UGA) for diagnostic evaluation. Other species involved in this outbreak from which H5 IAV was detected include two American crows (*Corvus brachyrhynchos*), one ruddy turnstone, eight horned grebes (*Podiceps auritus*), one peregrine falcon (*Falco peregrinus*), and one red-tailed hawk (*Buteo jamaicensis*) (Figure 1).

### 2.2. Postmortem Evaluation

Postmortem evaluation was performed at SCWDS on nine of the eleven submitted sanderling carcasses (W24-190A-E, G-J); evaluation of W24-190F and K was limited to external examination due to severe decomposition. Sex was determined by visualization of the gonads grossly and/or histologically and was considered undetermined when severe postmortem decomposition precluded identification of the gonads. Age determination was based on gross and/or microscopic presence (deemed juvenile) or absence (deemed adult) of a cloacal bursa, which regresses within one year post hatch in shorebirds [14]. Due to the collection time of year and highly variable preformative molt patterns in this species, age could not be determined solely from plumage [5]. Nutritional condition was subjectively deemed poor, fair, or good based on pectoral muscle robustness and grossly evident adipose in common depot sites (e.g., subcutis, overlying caudal coelomic viscera, coronary groove of heart, in the groove of the furcula). Oropharyngeal (OP) and cloacal (CL) swabs were collected from each of the eleven carcasses, pooled, and refrigerated prior to testing.

For histopathologic evaluation, samples included the pancreas, brain (cerebrum, midbrain, and/or cerebellum), kidney, heart, liver, lung, small and large intestines, and adipose from all individuals. In addition, the spleen, adrenal gland, gonad, trachea, esophagus, proventriculus, ventriculus, and skeletal muscle were included when available. Tissues were collected, fixed in 10% neutral buffered formalin, and routinely processed for histopathology (4-μm sections stained with hematoxylin and eosin) at the Histology Laboratory at UGA, Athens, GA, USA.

### 2.3. Immunohistochemistry

Interpretation of IAV IHC was challenged by postmortem changes, including freeze–thaw artifact and autolysis. Thus, IHC was not comprehensively performed across all tissues, but rather targeted brain, pancreas, and other lesioned tissues in eight of the nine sanderlings that underwent histopathologic evaluation. Methods were performed in the same Histology Laboratory as mentioned above and followed those of [15] with modifications. These included dilution of the primary antibody (AB20841, Abcam, Waltham, MA, USA) to 1:500 and antigen retrieval was with Antigen Retrieval Citra Solution (HK086-9K, BioGenex, Fremont, CA, USA). A section of lung from a ferret experimentally infected with influenza A virus served as a positive control on all IHC runs. Labeling was subjectively assessed as weakly, moderately, or strongly positive in the cytoplasm, nuclei, and/or pyknotic debris, when relevant. Evaluation of the strength and distribution of labeling was variably limited by postmortem changes.

### 2.4. Molecular Testing

Pooled OP and CL swabs were each placed in 2.0 mL of sterile brain heart infusion viral transport media supplemented with antimicrobials as previously described [16] and tested for clade 2.3.4.4b H5 IAV RNA by real-time reverse transcription polymerase chain reaction (rRT-PCR) [17]. Viral RNA was extracted using a KingFisher magnetic particle processor and a MagMAX-96 AI/ND Viral RNA isolation Kit (Ambion/Applied Biosystems, Foster City, CA, USA) following a modified MagMAX-M protocol [18]. Extracted RNA from each sample was screened by rRT-PCR against primers specific for clade 2.3.4.4b H5 IAV. Samples with a cycle threshold value < 40 underwent confirmatory testing and genotyping at the NVSL, USDA, Ames, IA, USA.

## 3. Results

### 3.1. Field, Demographic, and Spatiotemporal Data

Among submitted sanderlings, two were deemed juvenile, three were adults, and six were of unknown age. Four were male, five were female, and two were of unknown sex (Table 1). All eleven sanderlings had initial detections of clade 2.3.4.4b H5 IAV via rRT-PCR testing that were confirmed and genotyped at the NVSL. The outbreak continued into mid-April 2024 in and around Virginia Beach and Norfolk and along the western shore of Chesapeake Bay. In addition, sanderlings from which H5 IAV was detected were reported in a similar time frame in Rhode Island, New Hampshire, and Massachusetts [8,19].

### 3.2. Postmortem Findings

Nutritional status was poor to fair (Table 1) and cloacal bursas were grossly indiscernible in all nine necropsied sanderlings. Externally, carcasses had a small amount of sand adhered to the plumage, and two had variable blood staining on the feathers (Figure S1). Gross findings were few but may have been obscured by postmortem changes (e.g., autolysis). One sanderling (A) had multifocal, pinpoint, red foci on the surface of the pancreas. Occasional but inconsistent findings included bile-distended gallbladders, diffusely pale livers and spleens, dark red lungs, and diffusely reticulated kidneys. All sanderlings had minimal upper alimentary tract contents limited to a small amount of grit in the proventriculus and/or ventriculus (with crop empty). Intestines contained scant to small amounts of tan to green, viscous fluid.

**Figure 2:**
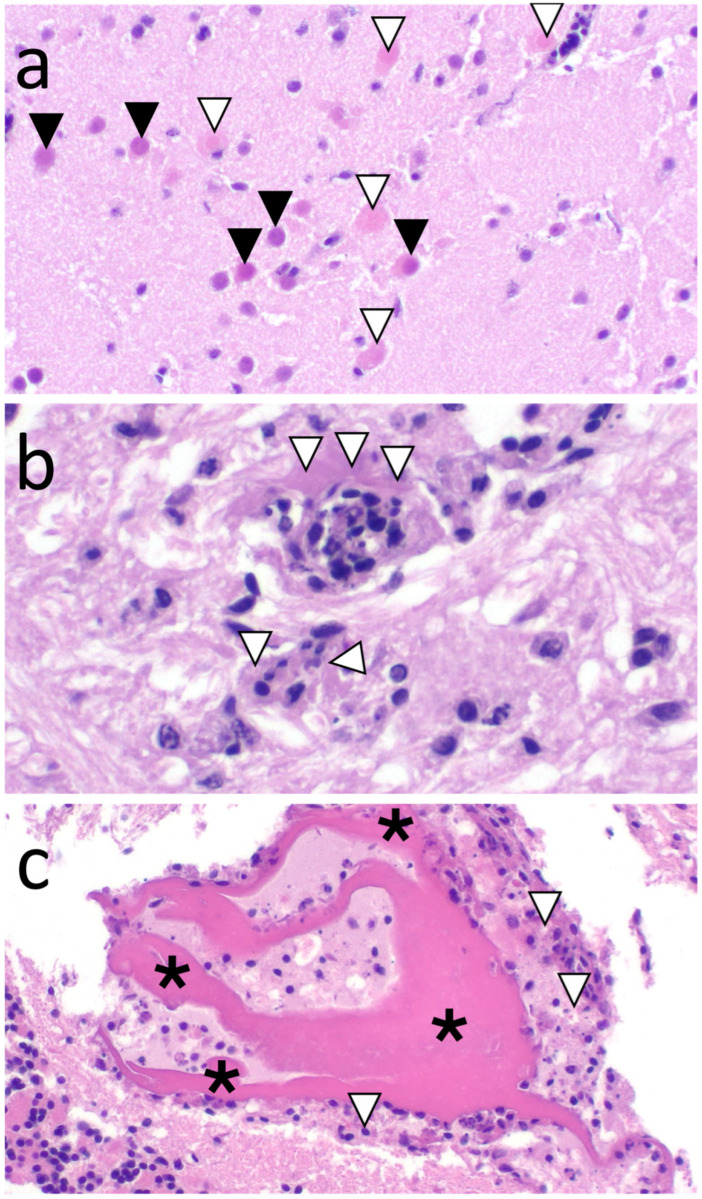
(**a**) Cerebral neuronal necrosis (white arrowheads) and intranuclear inclusions (black arrowheads) in sanderling W24-190E (magnification 20×); (**b**) fibrinoid necrosis (white arrowheads) in two arterioles in the midbrain in sanderling W24-190H (magnification 40×); (**c**) fibrinoheterophilic meningitis (asterisks—fibrin; white arrowheads—heterophilic inflammation) over the cerebellum of sanderling W24-190D (magnification 20×). (**a**–**c**) Hematoxylin and eosin stain.

**Figure 3:**
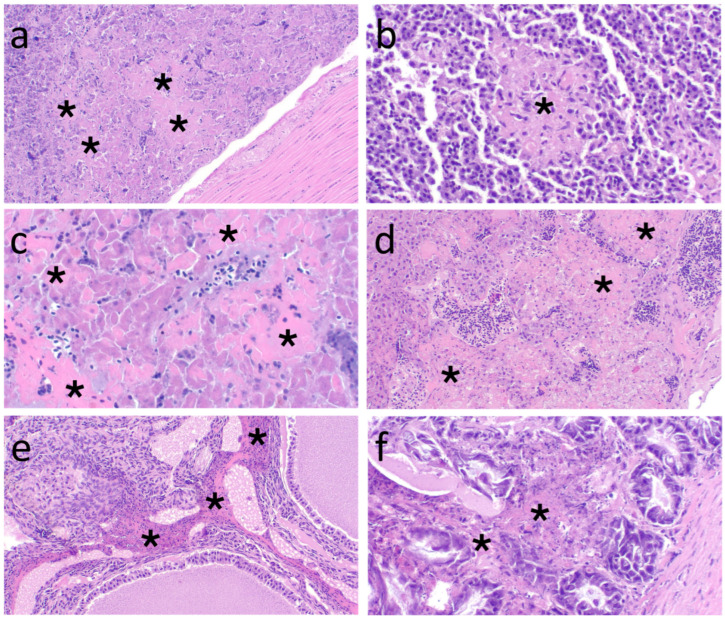
(**a**) Acute, multifocal, pancreatic necrosis (asterisks) with adjacent artery (lower right) in sanderling W24-190J (magnification 10×); (**b**) acute, focal, splenic necrosis (asterisk) in sanderling W24-190B (magnification 40×); (**c**) acute, multifocal, hepatic necrosis (asterisks) in sanderling W24-190J (magnification 20×); (**d**) acute, multifocal, adrenal gland necrosis (asterisks) in sanderling W24-190B (magnification 10×); (**e**) acute, multifocal, necroheterophilic oophoritis (asterisks) in sanderling W24-190B (magnification 10×); (**f**) acute, multifocal, small intestinal lamina propria necrosis (asterisks) with glandular atrophy and necrosis in sanderling W24-190-B (magnification 20×). (**a**–**f**) Hematoxylin and eosin stain.

The most notable histologic lesions based on severity and proportion of sanderlings affected were in the brain (Figure 2a–c) and pancreas (A, B, J) (Table 2; Figure 3a). The brain lesions were often evident in the cerebrum, midbrain, and cerebellum (A, B, D, E, G, H, I, J) and consisted of widely scattered necrotic neurons. Some neurons had prominent, magenta, homogenous, intranuclear presumed viral inclusion bodies (based on histologic appearance and IHC labeling; hereafter referred to as ‘inclusions’) (Figure 2a) that were most prominent in neuroparenchyma adjacent to ventricles and in the midbrain. Similar neuronal changes were evident in the spinal cord of the only sanderling in which this organ was evaluated (J). Few birds also had mild, lymphoplasmacytic perivascular cuffing (D) (Figure 2b); rare microgliosis (D, H, I, J); and/or superficial, necroheterophilic encephalitis with fibrinoid necrosis (D, H) and lymphoplasmacytic (J) or necroheterophilic (H) meningitis (Figure 2c). In the cerebellum, neuronal nuclear inclusions and neuronal necrosis were scattered throughout the molecular layer and segmentally in Purkinje cells (B, D, G, H, I). Pancreatic lesions were in all evaluated sanderlings for which autolysis was not too severe (A, B, J), and frequently consisted of severe, acute, multifocal to coalescing necrosis (Figure 3a). Less common lesions included: mild, multifocal splenic necrosis and/or lymphocytolysis in three sanderlings (B, D, J) (Figure 3b); mild, multifocal, renal tubular necrosis (E); moderate, multifocal renal tubular urate stasis (E, H); marked, widely scattered, hepatocellular necrosis with focal hemorrhage (J) (Figure 3c); severe, multifocal, bilateral adrenal necrosis (B; Figure 3d); and severe, ovarian stromal necrosis (Figure 3e) with intranuclear inclusions within mononuclear cells (B). Two sanderlings had gastrointestinal tract lesions, which included marked, multifocal, ulcerative, and necroheterophilic enteritis (Figure 3f), and colitis with hemorrhage (B) and focally extensive, marked, necroheterophilic, fibrinous serositis (A). Adipose lesions included moderate, focal, pericardial adipose necrosis (B); mild, multifocal, interstitial, peritracheal adipose necrosis with few mononuclear cells (C); and serous atrophy in pericardial fat (A), pericloacal fat (G), and bone marrow (E).

Incidental histologic findings included small intestinal luminal cestodes, large intestinal luminal metazoan eggs and pulmonary osseous metaplasia (A); focally extensive proventricular periglandular necrosis with glandular shrinkage or dilation with rare adult nematodes (C); pericloacal nematode-associated heterophilic granuloma (D); intraluminal (C, D, E, I) and mucosal (D only) adult cestodes in the small intestine; and segmental adult cestodes in small intestinal lumens (E, I). Diffuse vascular congestion was common in the heart and lung.

### 3.3. Immunohistochemistry

**Figure 4:**
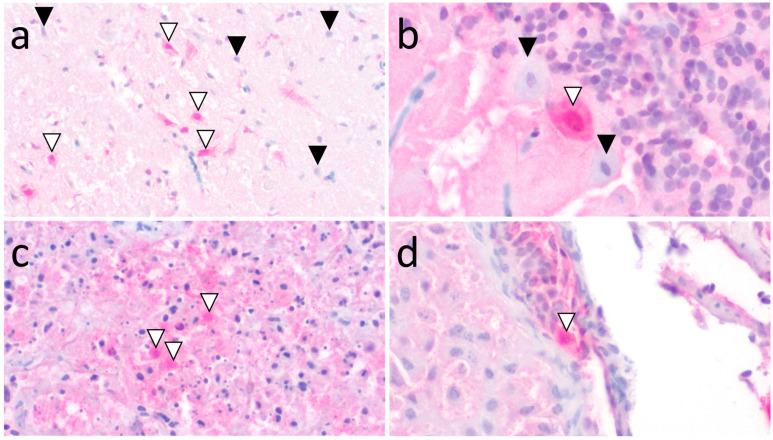
(**a**) Strong intranuclear and moderate intracytoplasmic neuronal IHC labeling (white arrowheads) in the midbrain of sanderling W24-190B (magnification 20×). Black arrowheads denote nonlabelled neurons for comparison; (**b**) strong intranuclear and moderate cytoplasmic Purkinje cell labeling (white arrowhead) in the cerebellum of sanderling W24-190G (magnification 40×). Black arrowheads denote nonlabelled neurons for comparison; (**c**) strong nuclear acinar epithelial cell labeling (white arrowheads) in the necrotic pancreas of sanderling W24-190A (magnification 20×); (**d**) strong nuclear and cytoplasmic labeling of a mononuclear cell (white arrowhead) along the edge of ovarian stroma in sanderling W24-190D (magnification 40×). (**a**–**d**) Immunohistochemistry for influenza virus A.

All eight sanderlings that underwent IHC of one or more tissues had labeling in the brain (8/9) and/or pancreas (4/4) (Table S1). In the brain, moderate to strong positive nuclear labeling, and less commonly concurrent moderate cytoplasmic labeling, was predominantly within widely scattered individual to regional clusters of neurons in the cerebrum and midbrain and within Purkinje cells in the cerebellum (Figure 4a,b). In the pancreas, moderate to strong positive cytoplasmic labeling was within variably sized groups of necrotic acinar epithelial cells (Figure 4c); however, severe necrosis and/or autolysis often involved rare discernible cells or cell remnants, which likely diminished visualization of intracellular influenza antigen. Rarely, moderate to strong positive nuclear labeling was also in ovarian stromal cells in 2/4 sanderlings and within dozens of widely scattered mononuclear cells in the spleen of another sanderling (Figure 4d). Tissues evaluated with rare to lack of evident influenza antigen immunolabeling included heart (0/8; nonspecific labeling was abundant), kidney (0/7), small and/or large intestine (0/8), lung (0/5), skeletal muscle (0/3), proventriculus (0/2), ventriculus (0/3), and spleen (1/3).

### 3.4. Molecular Testing

Pooled OP and CL swabs from all eleven sanderling carcasses yielded clade 2.3.4.4b H5 IAV RNA detections via rRT-PCR. H5 IAV RNA detections in other species that died during this outbreak included one ruddy turnstone, eight horned grebes, one peregrine falcon, and one red-tailed hawk. The pathogenicity and genotype of each virus was confirmed as HP H5 IAV at the NVSL, specifically EA/AM 2.3.4.4b H5N1 genotype B3.6 (NVSL Accession numbers 24-009941 and 24-009658).

## 4. Discussion

Shorebirds are a widely distributed and taxonomically diverse group that occupies a range of habitats and latitudes, owing in part to their capacity as long-distance migrants. While sanderlings are generally not considered to be under global conservation threat, climate change, habitat loss, resource depletion, human disturbance, and environmental contaminants cause concern in some parts of their range [5]. Given the ever-changing nature of these external factors, a clearer understanding of the threat of emerging pathogens, such as HP H5 IAV, on migratory shorebird species like the sanderling is urgently needed. HPAI is a devastating disease that has recently impacted many wild bird species that reside in and/or migrate across North and South America [7,8,11] and even Antarctic regions [9]. Thus, knowledge of the species-specific characterization of mortality events and pathology is critical to monitoring the ongoing impacts of HP H5 IAV on species and populations.

The geographically varied migration routes of sanderlings and the frequency with which they intermix with other species at stopover and destination sites [2,20] may facilitate IAV transmission, diversification, and movement. However, IAV detections at some migratory stopover sites in the early 2000s were rare in sanderlings, which have a lower IAV prevalence compared to that documented in other shorebird species [2,3,10]. Life history aspects such as roosting preferences (e.g., remote sandy points and islands) that entail limited interspecies mixing may lessen opportunities for IAV infection in sanderlings and some other shorebird species, in which infections are often attributed to local spillover events [2]. Unfortunately, avian species with lower exposure rates to LP IAVs may be susceptible to more severe disease from novel HP IAV infections due to a lack of pre-existing homo- or heterologous immunity [21].

As for numerous other wild bird species in the Western Hemisphere [7,11,12,15,22,23], our work shows that sanderlings are susceptible to acute and fatal HPAI. Sanderlings were previously reported among mass die-offs of wild seabirds and mammals attributed to HP IAV in November 2022 at multiple sites along the Peruvian coast [7]. During these outbreaks, sampled individual birds that exhibited one or more clinical signs (i.e., ataxia, circling, nystagmus, torticollis, disorientation, and dyspnea) or were found dead included a sanderling, Belcher’s gull (*Larus belcheri*), Humboldt penguin (*Spheniscus humboldti*), two Guanay cormorants (*Phalacrocorax bougainvillii*), and four Peruvian pelicans (*Pelecanus thagus*) [7]. HP IAV transmission to sanderlings in those die-offs was surmised to have occurred locally based on the rapid development of morbidity and mortality, placing doubt on their potential to move HP IAVs during migration [7]. HP IAV outbreaks in South America have been primarily identified as B3.2 genotype viruses, presumably introduced there by migrating wild birds after the initial incursion and westward expansion of A1 viruses (i.e., genotype of HP H5 IAV incursion into the Northeastern USA from Eurasia) following reassortment with LP IAVs [17,24]. Further reassortment events with North American LP IAVs gave rise to the B3.6 genotype, which was detected in sanderlings and other wild bird species in our study. However, as for those South American coastal die-offs, the total numbers of wildlife species with HPAI in our study are unknown and detections underrepresent the impact of these outbreaks. On both continents, sick and dying birds along coastlines create opportunities for spillover transmission events, such as have occurred in numerous predatory and scavenging wild mammals and birds [15,25], with potential for human infections [26,27].

Common challenges to free-ranging wildlife diagnostic evaluations, including the present study, include prolonged postmortem intervals and freeze–thaw cycles, which may obscure gross and histologic lesion patterns, immunohistochemical labeling, as well as viral detection. Most sanderlings in our study were in poor to fair nutritional condition at the time of death, which based on the acute lesion pattern, engenders the question of whether they were malnourished prior to HP H5 IAV infection and if this contributed to susceptibility or pathogenesis. The challenges of adequate resource acquisition and energetic demands of spring migration during the time leading up to HP H5 IAV infection may have further expedited disease development. This contrasts HPAI cases in North American raptors, waterfowl, and seabirds, many of which were in good nutritional condition at the time of death [28,29].

In conjunction with available shorebird data and those reported here in sanderlings, other recently reported histopathology findings among a broader scope of North American wild birds that died of HP H5 IAV will assist in understanding its varied pathogenesis and possibly species susceptibility. Sanderling pathology in our study most commonly included regional to widespread neuronal necrosis, often with intranuclear viral inclusions, which affected the cerebrum, midbrain, and cerebellum. Necrotic neurons were often in neuroparenchyma adjacent to the ependyma lining the ventricles. Less commonly, there was perivascular, lymphoplasmacytic encephalitis and microgliosis. When not masked by autolysis, the pancreas consistently exhibited severe, widespread necrosis. Among numerous North American bird groups, this pattern most resembles that of numerous migratory waterfowl species, like the Canada goose (*Branta canadensis*), Ross’ goose (*Anser rossii*), snow goose (*Anser caerulescens*), wood duck (*Aix sponsa*), red-breasted merganser (*Mergus serrator*), lesser scaup (*Aythya affinis*), trumpeter swans (*Cygnus buccinator*), and others [15,28]. In contrast, pancreatic lesions are less frequent and severe, and brain lesions are more variable (sometimes absent) in some North American raptor and galliform species [12,22,29].

The documentation of HP IAV-associated pathology in shorebird species is currently limited. Experimentally reproduced HP IAV H5N1 (A/whooper swan/Mongolia/244/05) infection and disease in a congeneric shorebird species, the dunlin (*Calidris alpina*), revealed differing histopathology patterns to naturally infected sanderlings in our study [30]. However, like sanderlings, dunlins proved susceptible to fatal HPAI. Inoculates exhibited fluffed feathers, appetite loss, ataxia, tremors, and motor control deficits and died within 5 days of inoculation. No gross pathology was observed, and histopathology was limited to mild lymphoplasmacytic meningitis with perivascular cuffing in the brain (11/23) and segmental Purkinje cell loss in one dunlin [30]. Unlike in sanderlings, no lesions were evident in the pancreas, gastrointestinal tract (esophagus, small intestine, ceca), kidney, adrenal gland, ovary, or testes of dunlins. Another HP IAV H5N1 (A/turkey/Turkey/1/2005) experimental study in red knots (*Calidris canutus islandica*) yielded similar clinical signs to dunlins, including sudden onset of lethargy, ataxia, and tremors; death or euthanasia occurred from 5 to 11 days post inoculation [31]. And while only 1/26 inoculated red knots had gross lesions (limited to diffuse, acute pancreatic vascular congestion), histopathologic lesions were more frequent and severe than in dunlins. The brain was the most commonly affected organ in red knots and lesions included multifocal, severe, acute encephalitis with perivascular cuffing, neuronal chromatolysis, neuronophagia, gliosis, rarefaction, and Purkinje cell loss. One clinically affected red knot also had multifocal, acute, necrotizing, and heterophilic pancreatitis. Brain and pancreatic lesions in naturally infected sanderlings in our study were more common, severe, and acute than in experimentally infected dunlins and red knots, with prominent, regional to widespread neuronal necrosis and intranuclear inclusions.

Similar to histopathologic patterns, knowledge of the distribution of IAV IHC labeling in sanderlings and other wild birds expands our understanding of virulence and pathogenesis, with the brain being a common target in many species. In raptors, HP H5 IAV is neuro-, epithelio-, and cardiomyotropic as demonstrated by the abundance of viral antigen labeling within these cell types (including neurons in the brain) as well as the reproductive tract [12,23,29,32]. Further, HP H5 IAV infection in skua and gull species in the United Kingdom is neuro-, pneumo-, lymphoid-, and cardiomyotropic according to histopathology and IHC labeling, with pancreatic and splenic necrosis as the most common lesions, which exhibited widespread immunolabeling. Neuronal necrosis and inflammation and necrosis in the reproductive tract also were reported [28]. In the Netherlands, sandwich terns (*Thalasseus sandvicensis*) with HPAI had pancreatic, duodenal, and pulmonary or nasal necrosis with corresponding IHC labeling [33]. In red knots, antigen labeling was often nuclear (and less commonly cytoplasmic) in neurons, glial cells, and/or ependymal cells and less common in other tissues (e.g., pancreatic exocrine cells, macrophages in liver and spleen) [31]. Immunolabeling in experimentally infected dunlins was consistent in the nuclei and cytoplasm of neurons and glial cells in the brainstem, and less consistent in other areas of the brain and other tissues (including the spleen, adrenal gland, cloacal bursal epithelium, intestine, ceca, and throughout the respiratory tract) [30]. Similar to histopathologic patterns, IHC labeling in the above species bears similarities to sanderlings, in which labeling in the brain corresponded to necrotic neurons (more commonly in nuclei but also in cytoplasm). Pancreatic labeling was less commonly observed and included degenerated and pyknotic cell components within necrotic foci. Similar to raptors, waterfowl, and other shorebirds, widespread lesion distribution (often necrosis) occurred in sanderlings, albeit less commonly, and was in the spleen, kidney, liver, adrenal gland, ovary/testis, and small and large intestines, with corresponding IHC labeling in most of those tissues.

The paucity of pathology and diagnostic data on shorebirds, including those with natural HP IAV infection, is likely in part attributable to logistical challenges to carcass sightings and collection, and to storage and delivery of diagnostically viable specimens to the laboratory. In sanderlings, mortality has been attributed to predation by raptors and hypothermia in nestlings [5]. Those causes can be presumed or confirmed by field observations, while diagnosis of infectious diseases generally requires both pathologic evaluation and laboratory testing. Infectious diseases documented in sanderlings include dermatophilosis (closely matched to the bacterium, *Dermatophilus congolensis*) in Louisiana and poxvirus in Florida [34,35], while the bacteria *Mergibacter septicus* (formerly Bisgaard taxon 40), *Salmonella* spp., and *Staphylococcus aureus*, and the fungus *Aspergillus* spp. have led to mortality in other shorebird species (e.g., terns, skimmers, and gulls) [36,37,38,39].

## 5. Conclusions

Our documentation of sanderlings with HPAI, combined with prior reports of infectious diseases in these and other shorebirds, highlights the critical need for health monitoring and diagnostic investigations in these birds, especially during mortality events. As long-distance migrants that traverse every continent and have a history of exposure to IAVs, sanderlings serve as a vital sentinel group for global health. Further work to assess the impacts of HP IAV in sanderlings and other migratory birds is needed, and should include seroprevalence and serologic responses, migration ecology of infected individuals, and experimental infections. The recent introductions, rapid spread, and devastating effects of HP H5 IAV on wild birds throughout North and South America and Antarctica underscores the critical demand for a better understanding of population status, risks, and management options for infectious disease challenges that overlay ongoing and persistent environmental and anthropogenic threats [5]. Concerns are further heightened by the threat of cross-species pathogen transmission and spillover, which can pose serious health risks to humans, domestic animals, and wildlife. These threats serve as a stark reminder of our limited control over and the fragility of complex ecological systems.

## Figures and Tables

**Table 1:** Demographic, weight, nutritional condition, and spatiotemporal data on eleven sanderlings diagnosed with highly pathogenic H5N1 influenza A virus from the Virginia coast, USA, in March–April 2024.

Case Identifier	Date Found Sick/Dead	Age ^1^	Sex	Weight (g)	Nutritional Condition	County	Coordinates (Latitude)	Coordinates (Longitude)
W24-190A	13 March 2024	U	Female	32.5	Poor	Virginia Beach	36.91926	−75.9941
W24-190B	10 March 2024	U	Female	37.1	Poor	Virginia Beach	36.92009	−76.13264
W24-190C	9 March 2024	Adult	Male	38.6	Poor	Norfolk	36.94190	−76.22831
W24-190D	9 March 2024	Juvenile	Female	47.5	Fair	Norfolk	36.94190	−76.22831
W24-190E	9 March 2024	U	U	39.2	Fair	Norfolk	36.94190	−76.22831
W24-190F	7 March 2024	U	Female	54.1	Fair	Hampton	37.04608	−76.28682
W24-190G	8 March 2024	Adult	Female	42.3	U	Norfolk	36.96474	−76.26736
W24-190H	8 March 2024	Adult	Male	39.7	Fair	Norfolk	36.93314	−76.20011
W24-190I	12 March 2024	U	U	32.9	Poor	Norfolk	36.96919	−76.29353
W24-190J	11 March 2024	Juvenile	Male	37.7	U	Norfolk	36.95553	−76.52336
W24-190K	Not reported	U	Male	48.5	Fair	U	U	U

^1^ U—unknown or not reported.

**Table 2:** Histopathology scores by tissue type of nine sanderlings diagnosed with highly pathogenic influenza from the Virginia coast, USA, in March–April 2024 (W24-190F and W24-190K were not examined) ^1–3^.

ID	Pancreas	Cerebrum	Midbrain	Cerebellum	Spleen	Kidney	Liver	Adrenal Gland	Gonad	Small Intestine	Large Intestine
W24-190A	++++ (N,HI)	++++ (N,NI)	NSL	NE	NE	NSL	NSL	NE	NSL	NSL	++ (N,HI)
W24-190B	++++ (N)	+++ (NI)	++ (NI)	+++ (NI)	++ (N)	NSL	NSL	++++ (N)	+++ (N)	++ (N,H,U)	+++ (N,HI,H)
W24-190C	SA	NE	NE	NE	NSL	NSL	NSL	NE	NSL	NSL	NSL
W24-190D	SA	+++ (N,PVC)	+++ (NI,PVC)	+++ (N,G,FN)	++ (N)	NSL	NSL	NSL	NSL	NSL	NSL
W24-190E	NE	+++ (N,NI)	NE	NSL	NE	++ (N)	NSL	NE	NE	SA	SA
W24-190G	SA	NSL	+++ (N)	++ (NI)	NE	NSL	SA	NSL	NSL	NSL	NSL
W24-190H	SA	NSL	+++ (N,HI,FN,G)	++ (N,HI,M)	NE	NSL	NSL	NE	NE	SA	NE
W24-190I	SA	+++ (N,NI,G)	NE	+ (NI)	NSL	NSL	NSL	NE	NE	SA	NSL
W24-190J	++++ (N)	++ (NI,M)	++ (N,NI,G)	NSL	++ (L)	NSL	+++ (N,H)	NE	NE	NSL	NE
Proportion (%) affected	3/3 (100%)	6/8 (75%)	5/6 (83%)	5/6 (83%)	3/5 (60%)	1/9 (11%)	1/8 (13%)	1/3 (33%)	1/5 (20%)	1/6 (17%)	2/6 (33%)

^1^ No significant lesions were evident in heart (*n* = 9); lungs (*n* = 8); skeletal muscle (peritracheal or pectoral; *n* = 5); ventriculus (*n* = 3); proventriculus and trachea (*n* = 2 each); esophagus (*n* = 1); nor in opportunistically evaluated great vessels, peripheral nerves, uropygial gland, oviduct, and skin. Adipose had foci of necrosis in sanderlings W24-190B (+++) and W24-190C (+); sanderlings W24-190A, E, and G had fat atrophy. ^2^ Subjective lesion scoring included mild (+), moderate (++), marked (+++) and severe (++++). ^3^ N—necrosis; HI—heterophilic inflammation; SA—severe autolysis (precluding accurate evaluation); NSL—no significant lesions; NI-nuclear neuronal inclusions; NE—not examined; PVC—periventricular; G—microgliosis; FN—fibrinoid necrosis, M—meningeal involvement; L—lymphocytolysis; H—hemorrhage. Those with SA were not included in denominators.

## Data Availability

The original contributions presented in this study are included in the article/Supplementary Materials; further inquiries can be directed to the corresponding author.

## Supplementary Materials

The following supporting information can be downloaded at: https://www.mdpi.com/article/10.3390/ani15142057/s1: Figure S1: (a) Eight of the evaluated sanderlings from an HP IAV mortality event in Virginia in 2024; most carcasses exhibited moderate to marked postmortem change and were in fair to poor nutritional condition; (b) note the diffusely pale liver (asterisk) and spleen (white arrowhead) in sanderling W24-190G. For anatomic reference, a black arrowhead denotes the heart, and a caret denotes the ventriculus; Table S1: Immunohistochemistry results of nine sanderlings with natural highly pathogenic avian influenza virus infection in Virginia, USA, in March–April 2024. “Absent” refers to the absence of labeling.

## Abbreviations

The following abbreviations are used in this manuscript:

USA	United States of America
HP	Highly pathogenic
LP	Low pathogenic
IAV	Influenza A virus(es)
NVSL	National Veterinary Services Laboratory
USDA	United States Department of Agriculture
HPAI	Highly pathogenic avian influenza (disease)
IHC	Immunohistochemistry
SCWDS	Southeastern Cooperative Wildlife Disease Study
UGA	University of Georgia
A1	Genotype of HP H5 IAV incursion to the Northeastern USA from Eurasia
OP	Oropharyngeal
CL	Cloacal
rRT-PCR	Reverse transcription polymerase chain reaction
RNA	Ribonucleic acid
EA/AM	Reassortant of H5 goose/Guangdong and North American wild bird lineage
