# Supplementary material for: Clade 2.3.4.4b Highly Pathogenic Avian Influenza H5N1 Pathology in a Common Shorebird Species (Sanderling; Calidris alba) in Virginia, USA
Source: Animals (Basel). 2025 Jul 12;15(14):2057. doi: 10.3390/ani15142057 (PMC12291917; doi:10.3390/ani15142057)
Supplement: Supplementary file 1 [file animals-15-02057-s001.zip › animals-3731627-supplementary.pdf]

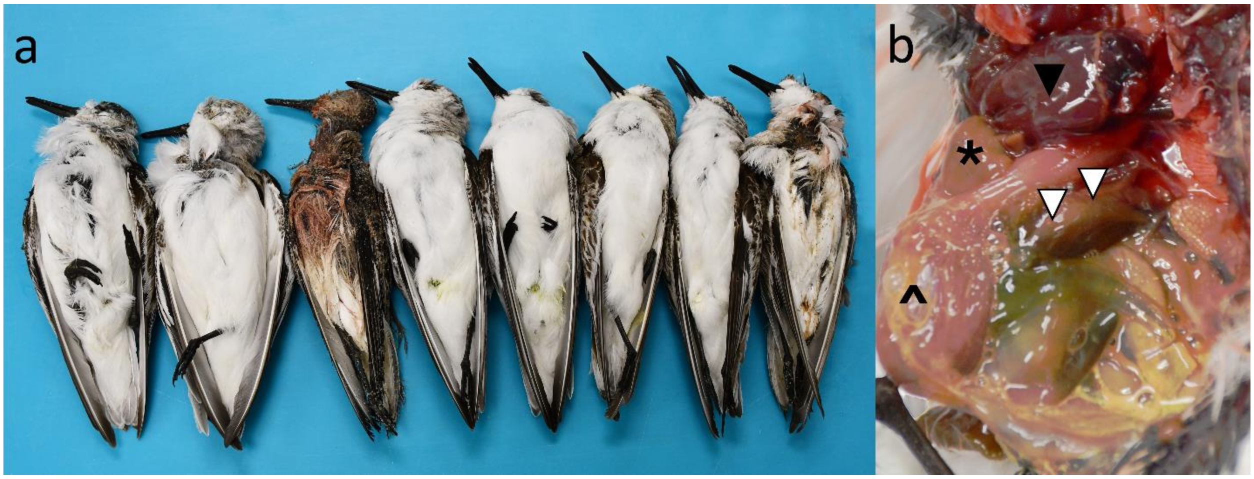

Figure S1: (a) Eight of the evaluated sanderlings from a HP IAV mortality event in Virginia in 2024; most carcasses exhibited moderate to marked postmortem change and were in fair to poor nutritional condition; (b) Note the diffusely pale liver (asterisk) and spleen (white arrowhead) in sanderling W24-190G. For anatomic reference, a black arrowhead denotes the heart, and a caret denotes the ventriculus.

Figure S1: (b) Original digital image.

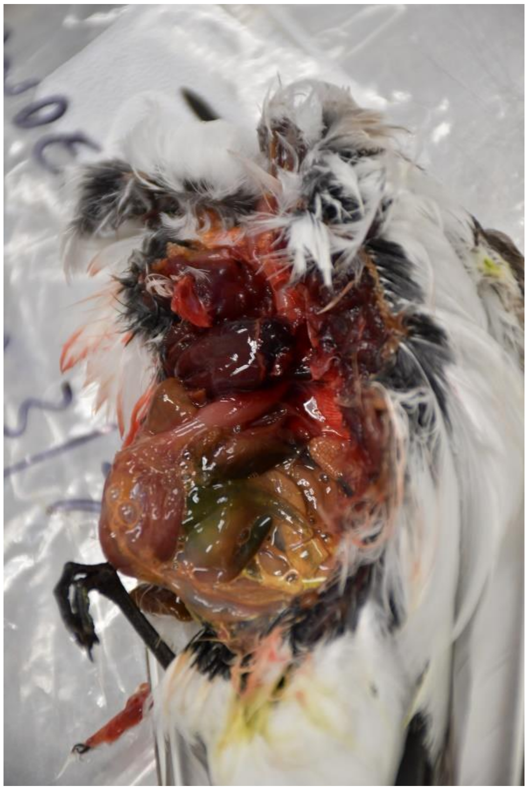

Table S1: Immunohistochemistry labeling intensity, cellular location and cell type in nine sanderlings with natural highly pathogenic avian influenza virus infection in Virginia, USA in March-April 2024 [1].

Case identifier	Pancreas	Brain	Adrenal gland	Ovary	Spleen	Heart	Lung	Liver	Kidney	Provent-riculus	Ventric-ulus	Intest-ine	Skel-etal muscle
W24-190A	+++; moderate to strong ICIN necrotic acinar cells	+++; Cerebrum and midbrain: hundreds, widely scattered and in loose clusters, strong INIC neurons; Cerebellum: absent (small section evaluated)	NE	+; rare IN stromal cells	NE	Absent	Absent	NE	Absent	NE	Absent	Absent	NE
W24-190B	+; moderate to strong ICIN necrotic acinar cells	++; Cerebrum: rare, widely scattered, strong IN neurons; Midbrain: multifocal clusters of IN neurons; brainstem IN neurons near cerebellar junction	NE	NE	NE	Absent	NE	NE	NE	Absent	Absent	Absent	NE
W24-190D	+; moderate to strong ICIN necrotic acinar cells	Absent	+; rare IC interrenal cells	+; rare IN stromal cells	NE	Absent	Absent	NE	Absent	NE	NE	Absent	NE
W24-190E	NE	++; Cerebrum: moderate, regional, dozens, IN neurons	NE	NE	NE	Absent	Absent	NE	Absent	Absent	Absent	Absent	Absent
W24-190G	NE	++; Cerebrum: few small clusters strong INIC neurons; Cerebellum: strong IN Purkinje cells, dozens, widely scattered	NE	Absent	NE	Absent	NE	Absent	Absent	NE	NE	NE	Absent
W24-190H	NE	++; Midbrain and cerebrum: strong INIC neurons, single, widely scattered, dozens; cerebellum: absent	NE	Absent	Absent	Absent	Absent	Absent	Absent	NE	NE	NE	Absent

Case identifier	Pancreas	Brain	Adrenal gland	Ovary	Spleen	Heart	Lung	Liver	Kidney	Proventriculus	Ventriculus	Intes-tine	Skel-etal muscle
W24-190I	SA	++; Cerebrum: strong IN neurons, dozens, multiregional; cerebellum: few, rare IN neurons (including Purkinje cells)	NE	NE	Absent	Absent	NE	Absent	Absent	NE	NE	Absent	NE
W24-190J	+; moderate to strong ICIN necrotic acinar cells	+; Midbrain: few, widely scattered, single IN neurons, moderate plus cluster w/stronger labeling; cerebellum: absent	NE	NE	+; moderate IN, dozens, mononuclear cells	Absent	Absent	Absent	Absent	NE	NE	Absent	NE
Proportion (%) affected	4/4 (100%)	8/9 (89%)	1/1 (100%)	2/4 (50%)	1/3 (33%)	0/9	0/5	0/4	0/7	0/2	0/3	0/6	0/3

[1] IC, intracytoplasmic; IN, intranuclear; NE, not examined; SA, severe autolysis (precluding accurate evaluation and thus not included in denominators); "Absent" refers to absence of labeling.
